# Some Homeopathic Therapeutics

**Published:** 1894-06

**Authors:** 


### *EDITORIAL.*

The office of THE JOURNAL is in room 330 Equitable Building.

Address all communications, and make all remittances payable to THE ATLANTA MEDICAL AND SURGICAL JOURNAL, Atlanta, Ga.

Articles for publication should reach this office not later than the 15th instant.

The editors are not responsible for views expressed by contributors.

### SOME HOMEOPATHIC THERAPEUTICS.

Persons who do not have occasion to read homeopathic journals can have no idea of the ridiculous *tommy-rot* that they sometimes contain with respect to the therapeutic indications of drugs.

We learn from a late issue of the *Hahnemannian Monthly* that if a patient sleeps with knees apart give him chamomile; if he sleeps with legs stretched out at full length give pulsatilla; and if he sleeps with one leg drawn up and the other stretched out give stannum; if a patient has a hot, pale cheek and a cold, red cheek give moschus; if a patient in confinement "curses" you, spits in your face and pulls your whiskers give chamomile, etc.

The *North American Journal of Homeopathy* for May contains a very exhaustive study entitled "Some Peculiar Sweats." The treatment of them is equally as peculiar as the sweats. Thus if only the front part of the body sweats, *amanita*, *phosphorus*, etc.; if the back part of the body, *sulphur;* if only the upper half of the body sweats, *nux vomica*, *opium*, etc.; if the lower half, *crocus*, *cyclamen*, etc.; if one sweats on the right side, *aurum*, *sodium*, etc.; if he sweats on the left side, *fluoric acid* and *jaborandi;* if he sweats only on the face, *ignatia;* and finally, if he sweats in spots, *tellurium*. For sweats after coughing, *arsenic;* for sweats as soon as one sleeps, *arsenic;* for sweats as soon as he drops into a sound sleep, *cinchona;* for sweats in the morning, *angostura;* for sweats while eating, *oleum animalis;* for sweats after unsatisfactory coition, *jambos eugenia.* If the sweat has a sweet smell, *caladium;* if it smells sour, *natrum phosphas;* if it smells like spice, *rhododendron;* if it smells like horse's urine, *nitric acid.*

So also in the *Southern Journal of Homeopathy,* for February, there is an elegant paper on "Some Peculiar Coughs," with the indication for each variety. Thus, when the cough occurs every morning at six o'clock, *cedron;* when it occurs in the day, not at night, *ferrum;* when it occurs in the night, not in the day, *conium;* when the cough is worse when lying on belly, *baryta;* when worse when lying on left side, *mercurius;* when the cough is aggravated in the presence of strangers, *baryta;* when aggravated by music, *creasote;* when it comes from playing on the piano, *calcarea;* when the cough occurs regularly from February until summer, *stannum;* when involuntary stool occurs with the cough, *phosphorus;* when involuntary emission of urine occurs with it, *alumina, capsicum, bryonia,* and others.

And so on *ad nauseam.* We may remark, however, on the authors' originality, for we are sure many of the above peculiar sweats and coughs were never observed by others; as for these therapeutics, they doubtless represent the school very fairly. Their absurdity is sufficiently apparent, and cannot be heightened by comment.

